# Supplementary material for: Identification of distinct cytotoxic granules as the origin of supramolecular attack particles in T lymphocytes
Source: Nat Commun. 2022 Feb 24;13:1029. doi: 10.1038/s41467-022-28596-y (PMC8873490; doi:10.1038/s41467-022-28596-y)
Supplement: Supplementary file 1 — Supplementary Information [file 41467_2022_28596_MOESM1_ESM.pdf]

Identification of a novel class of cytotoxic granules as the origin of supramolecular attack particles in T lymphocytes

Hsin-Fang Chang,[1,9,10*] Claudia Schirra,[1,9] Momchil Ninov,[2,8] Ulrike Hahn,[1] Keerthana Ravichandran,[1] Elmar Krause,[1] Ute Becherer,[1] Stefan Balint,[3] Maria Harkiolaki,[4] Henning Urlaub,[2] Salvatore Valitutti,[5,6] Cosima T. Baldari,[7] Michael L. Dustin,[3] Reinhard Jahn,[8] and Jens Rettig[1*]

Supplementary data

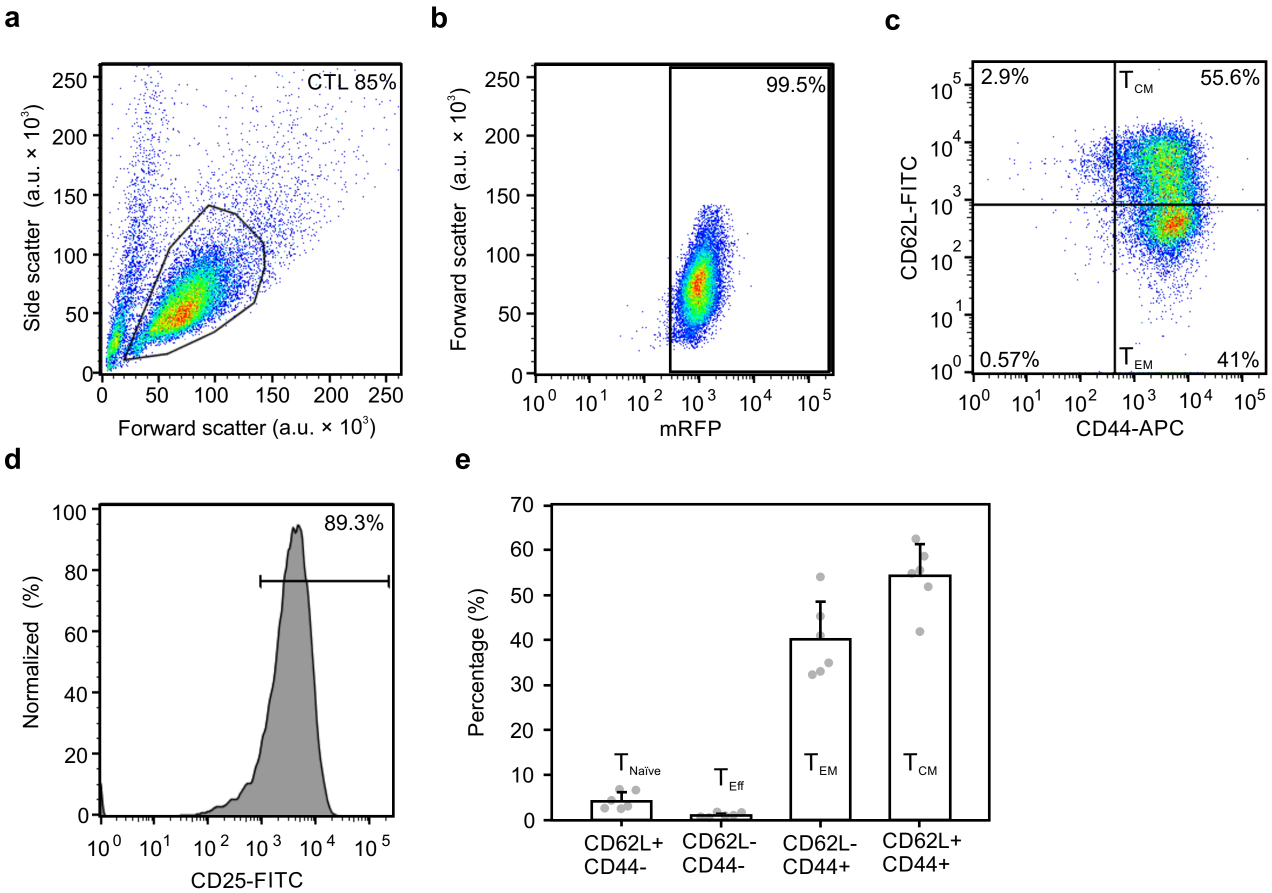

Supplementary Figure 1: Flow cytometric analysis of Syb2-mRFP positive CTL used for granule isolation. a, Percentage of living CTLs determined by granularity (side scatter) and size (forward scatter). **b,** Percentage of Syb2-mRFP positive CTLs. **c,** Representative experiment to show the percentage of naïve (T_{naive}, CD62L$^+$/CD44$^-$), effector (T_{EM}, CD62L$^-$/CD44$^-$), effector memory (T_{Eff}, CD62L$^-$/CD44$^+$) and central memory CTL (T_{CM}, CD62L$^+$/CD44$^+$) in the culture. **d,** Percentage of activated CTLs by detecting CD25 expression. **e,** Bar graph of the average percentages superimposed on a scatter dot plot (gray) of naïve, effector, central memory and effector memory lymphocytes in the Syb2-mRFP mouse CTL culture, three days after bead activation (N=6, values are given as mean ± sem). N = 6 independent experiments.

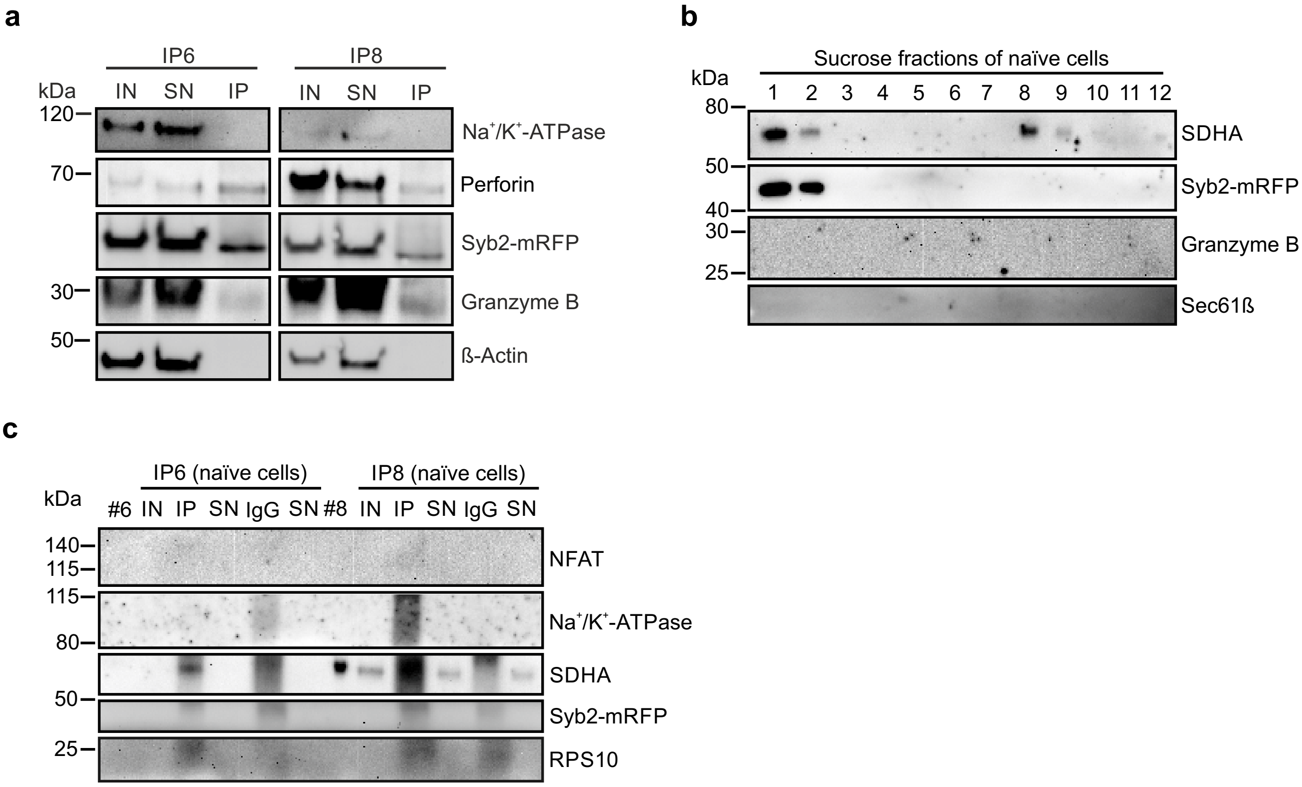

Supplementary Figure 2: Subcellular fractionation and immuno-isolation of CG from stimulated and naïve cells of SybKI mice. a, Western Blot of IP6 and IP8 from stimulated cells shown in Fig. 2b with 3% input (IN), 3% supernatant (SN), 3% immunoprecipitation (IP). Immunoblot analysis was done with different markers for plasma membrane (Na^+/K^+ATPase), cytotoxic granules (Syb2-mRFP, GzmB and Prf1), endoplasmic reticulum (Sec61β) and cytoskeleton (β-actin, reprobed). N=1. **b,** Non-quantitative western blot for sucrose fractions 1-12 derived from naïve CTL. 18 µl of each sample were separated on a 4-12% Bis/Tris gel with MOPS buffer. Immunoblot analysis was done with markers for mitochondria (SDHA), cytotoxic granules (Syb2-mRFP, GzmB) and endoplasmic reticulum (Sec61ß). N=1 **c,** Western blot of IP6 and IP8 from naïve CTL with 1% input (IN), 1% supernatant (SN), 20% of immuno-precipitate (IP) and 20% of immuno-precipitate with control IgG (IgG) volume on a 4-12% Bis/Tris gel with MOPS buffer. 18 µl of sucrose fractions 6 and 8 were separated in lane 1 and 7, respectively. Immunoblot analysis was done with different markers for T-cell activation (NFAT, reprobed), plasma membrane (Na^+/K^+-ATPase), mitochondria (SDHA), cytotoxic granules (mRFP), endoplasmic reticulum (Sec61ß) and proteome (RPS10). N=1.

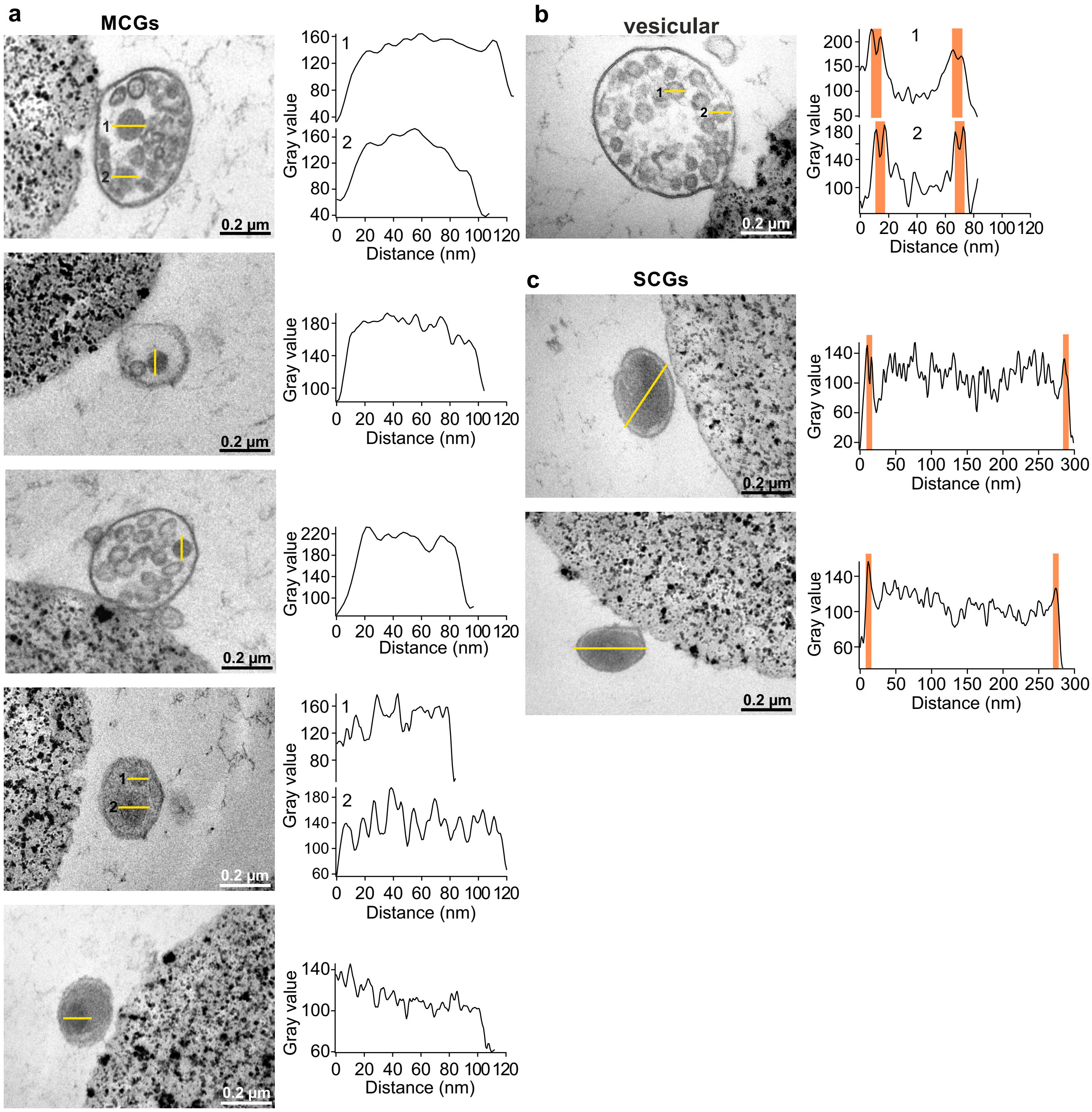

Supplementary Figure 3: Line profile analysis of SMAPs and small vesicles contained in MCGs.
a, Gallery of electron micrographs of MCGs (left panel) out of 118 granules from 3 experiments. Profile plot of dense cores without a typical lipid bilayer analyzed for Fig. 3e (right panel). Scale bar 0.2 µm. **b,** Electron micrograph of an exemplary multivesicular body with intraluminal vesicles characterized by their lipid bilayer membrane (left panel). Profile plot of two intraluminal vesicles (right panel). The orange bars mark their membrane. The polar heads of the lipid appear as electron-dense dark lines separated by a less-dense zone, corresponding to the aliphatic chain. Scale bar 0.2 µm. **c,** Two electron micrographs of SCGs (left panel) and their profiles plot (right panel) out of 85 granules from 3 experiments. The orange bars show the membrane with or without separated dense dark lines. The density profile was measured with ImageJ along 2-pixel-thick yellow lines shown on the representative micrographs. The images were inverted and filtered with a bandpass filter (2-100 pixels, ImageJ Plugin "FFT Bandpass Filter") before measurement.

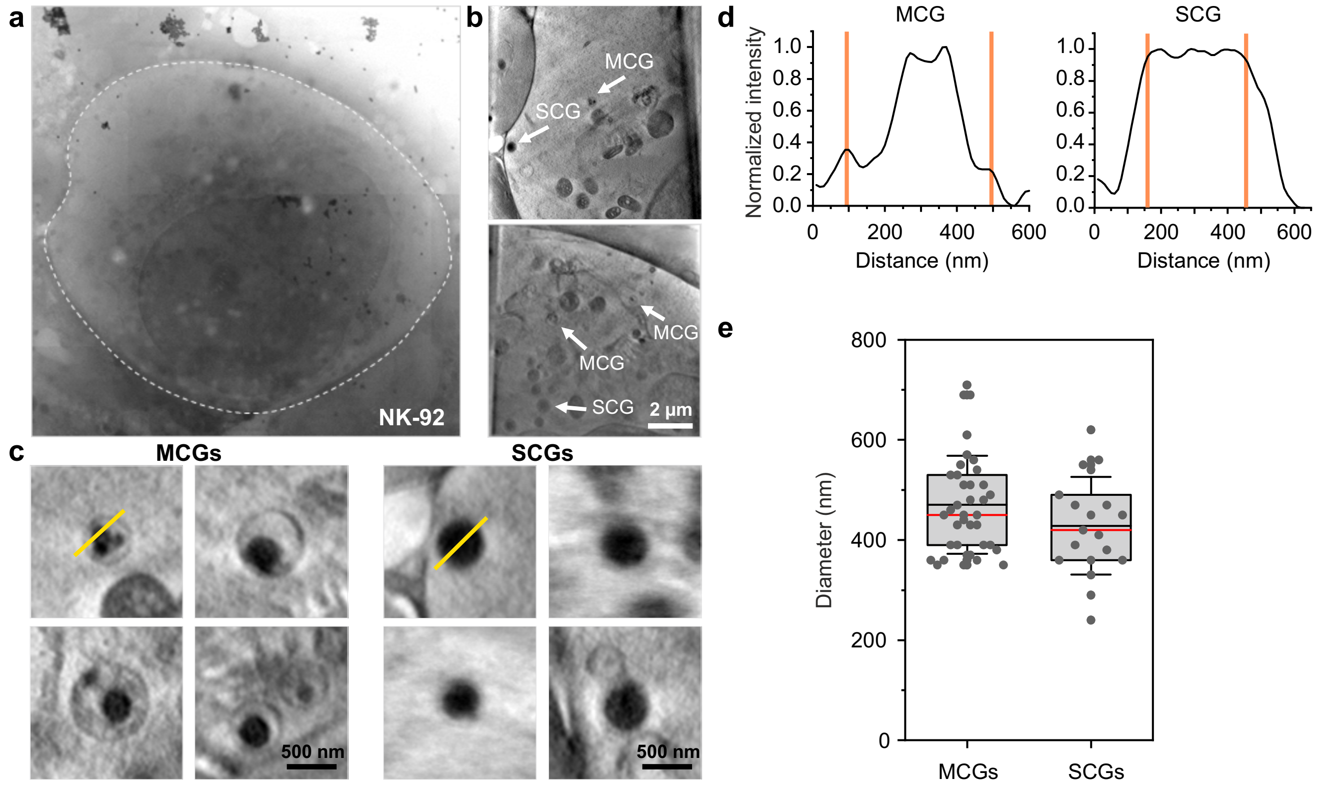

Supplementary Figure 4: Human NK cells contain MCGs and SCGs.

a, Cryo-Soft-X-ray image of an individual natural killer cell (NK92). **b**, Zoomed high resolution Cryo-Soft X-Ray image of SCGs and MCGs, including SMAPs in the NK92 cell in (**a**). **c,** Representative zoomed high resolution Cryo-Soft X-Ray images of individual SMAPs in MCGs and SCGs from NK92 cells. Scale bar 2 µm and 500 nm, respectively. **d,** Intensity profiles measured along the yellow line of one MCG (left) and one SCG (right) shown in (**c**). Note that the membrane is clearly visible as secondary peaks on the plot of the MCG but not in the SCG. **e**, Box plot superimposed with a scatter dot plot of SCGs and MCGs diameter from Cryo-Soft X-Ray images as shown in (**c**). The black line corresponds to the average diameter of 470 ± 98 nm and 428 ± 98 nm for MCGs and SCGs, respectively (mean ± SD). The red line represents the median diameter. Data are from N=37 and 19 MCGs and SCGs, respectively.

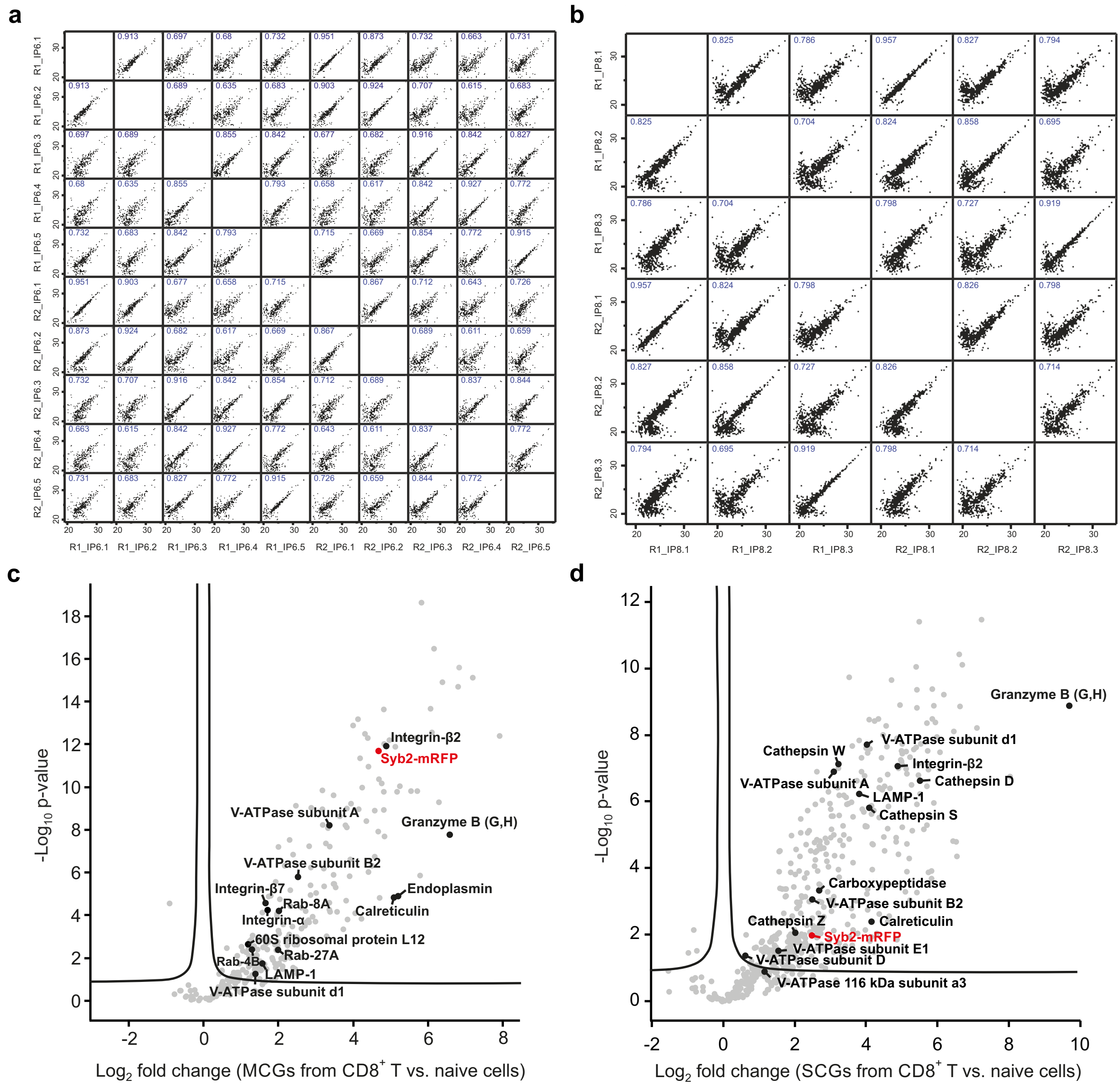

Supplementary Figure 5: Pearson's correlation of protein LFQ intensities and volcano plots of proteins in MCG and SCG. a, Five biological and technical replicates were compared with each other for IP6. The Pearson correlation gave R^2 values ranging between 0.611-0.951 showing a high reproducibility. **b,** For IP8 three biological and technical replicates were compared with each other (R^2 values 0.695-0.957). **c,** Shows the –Log p-values of proteins contained in MCGs of activated CTLs against naïve cells. Points above cutoff curves are significantly abundant proteins. SMAP-associated (e.g. 60S ribosomal protein L12 (RPL12), HSP90b1) and cytotoxic granule proteins (e.g. Rab27a, Syb2 and GzmB) were significantly co-enriched with the antigen. **d,** Shows the –Log p-values of the proteins contained in SCGs of activated CTLs against naïve cells. In addition to Syb2, cytotoxic granule (CG) marker proteins such as GzmB and multiple cathepsin isoforms (W, D and S) were significantly enriched.

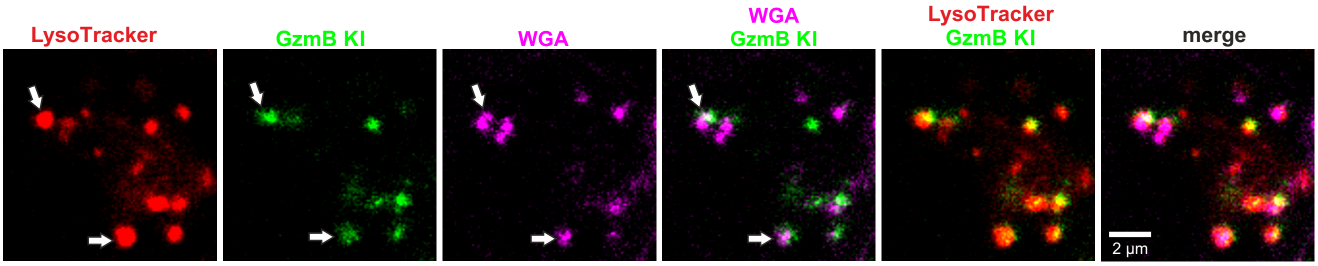

Supplementary Figure 6: MCG is an acid compartment.
Confocal single stack image of a live GzmB KI CTL that was loaded with LysoTracker red and WGA-647. White arrows point to MCGs that were co-labeled with GzmB and WGA. LysoTracker labeled both SCG and MCG. N=1, n=200. Scale bar, 2 µm.

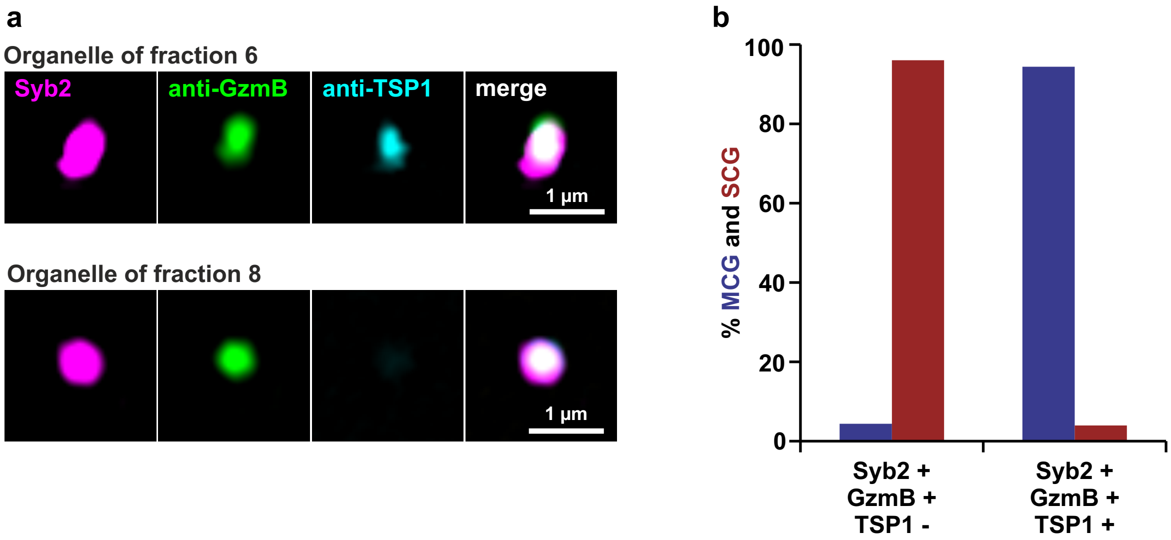

Supplementary Figure 7: Thrombospondin-1 is a specific marker for MCGs.
a, Representative single plane SIM images of isolated MCG (upper row) and SCG (lower row) of Syb2-KI mouse CTLs after coverslip centrifugation and immunostaining against GzmB (green) and TSP-1 (cyan) are shown. Scale bar, 1 µm. **b,** Percentage of Syb2 and GzmB positive MCGs and SCGs with and without endogenously expressed TSP-1. Experiments were done as described in (a); N=3, MCG: n=91 and SCG: n=73.
