# Supplementary material for: Identification of distinct cytotoxic granules as the origin of supramolecular attack particles in T lymphocytes
Source: Nat Commun. 2022 Feb 24;13:1029. doi: 10.1038/s41467-022-28596-y (PMC8873490; doi:10.1038/s41467-022-28596-y)
Supplement: Supplementary file 3 — Description of Additional Supplementary Files [file 41467_2022_28596_MOESM3_ESM.pdf]

Description of Additional Supplementary Files

File Name: Supplementary Data 1
Description: List of proteins of MCG and SCG from mass spectrometry analysis.

File Name: Supplementary Movie 1
Description: Cryo-Soft X-Ray tomography of SCGs from NK92 cells. White arrows pointed to individual SCGs.

File Name: Supplementary Movie 2
Description: Cryo-Soft X-Ray tomography of MCGs from NK92 cells. White arrows pointed to individual MCGs.

File Name: Supplementary Movie 3
Description: SCG and MCG secretion at the IS. CTLs isolated from GzmB KI mouse were stained with WGA-647 to label MCG. Cells were placed on the stimulated lipid bilayer with ICAM-1 and anti-CD3 antibody coating to induce synapse formation. Secretion from both classes of granules was recorded by TIRFM.

File Name: Supplementary Movie 4
Description: SMAP secretion from MCGs at the IS. CTLs were co-transfected with GzmB-mCherry and TSP-1-GFPspark. The synapse formation was induced by anti-CD3 coated glass coverslip. The granule secretion was recorded by TIRFM.
