# Supplementary material for: Identification of distinct cytotoxic granules as the origin of supramolecular attack particles in T lymphocytes
Source: Nat Commun. 2022 Feb 24;13:1029. doi: 10.1038/s41467-022-28596-y (PMC8873490; doi:10.1038/s41467-022-28596-y)
Supplement: Supplementary file 9 — Reporting Summary [file 41467_2022_28596_MOESM9_ESM.pdf]

nature research

Corresponding author(s): Hsin-Fang Chang & Jens Rettig

Last updated by author(s): 2021.12.07

Reporting Summary

Nature Research wishes to improve the reproducibility of the work that we publish. This form provides structure for consistency and transparency in reporting. For further information on Nature Research policies, see our Editorial Policies and the Editorial Policy Checklist.

Statistics

For all statistical analyses, confirm that the following items are present in the figure legend, table legend, main text, or Methods section.

n/a	Confirmed	
☐	☒	The exact sample size (n) for each experimental group/condition, given as a discrete number and unit of measurement
☐	☒	A statement on whether measurements were taken from distinct samples or whether the same sample was measured repeatedly
☐	☒	The statistical test(s) used AND whether they are one- or two-sided<br>*Only common tests should be described solely by name; describe more complex techniques in the Methods section.*
☐	☒	A description of all covariates tested
☒	☐	A description of any assumptions or corrections, such as tests of normality and adjustment for multiple comparisons
☐	☒	A full description of the statistical parameters including central tendency (e.g. means) or other basic estimates (e.g. regression coefficient) AND variation (e.g. standard deviation) or associated estimates of uncertainty (e.g. confidence intervals)
☐	☒	For null hypothesis testing, the test statistic (e.g. F, t, r) with confidence intervals, effect sizes, degrees of freedom and P value noted<br>*Give P values as exact values whenever suitable.*
☒	☐	For Bayesian analysis, information on the choice of priors and Markov chain Monte Carlo settings
☒	☐	For hierarchical and complex designs, identification of the appropriate level for tests and full reporting of outcomes
☐	☒	Estimates of effect sizes (e.g. Cohen's d, Pearson's r), indicating how they were calculated

Our web collection on statistics for biologists contains articles on many of the points above.

Software and code

Policy information about availability of computer code

Data collection	Data generated from confocal microscopy (Zeiss) and structured illumination microscopy (Zeiss) were collected using Zen 2011 software. FACS data were collected by BD FACSAriaIII analyzer (BD Biosciences) and analyzed using BD FACSDivaTM software 6.0. TIRF data were collected from Visiview (Visitron-Systems GmbH). STED images were acquired from Imspector software V16.3 (Abberior instruments). Electron microscope data were collected from Olympus iTEM 5.0 image software and microscope control software (version 7.0). The gel documentations for Western blots were acquired from FluorChem M system, ProteinSimple. Mass spectrometry data were collected by LC-MS/MS (Thermoscientific).
Data analysis	Detail description of data analysis are in methods section of the manuscript. Imaging data were analyzed by imageJ, Fiji (V15 and above) and Zen 2012 software. Cryo-Soft X-ray Tomography was analyzed with etomo, part of the IMOD package. FACS Data was analyzed using BD FACSDivaTM software. Mass spectrometry data are analyzed by MaxQuant (MQ) software (version 1.6.0.1) and Perseus (version 1.6.2.2). Statistic analysis and graphing was performed with Igor V6.37 (Wavemetrics) and SigmaPlot V13.0 (Systat Software, Inc.).

For manuscripts utilizing custom algorithms or software that are central to the research but not yet described in published literature, software must be made available to editors and reviewers. We strongly encourage code deposition in a community repository (e.g. GitHub). See the Nature Research guidelines for submitting code & software for further information.

Data

Policy information about availability of data

All manuscripts must include a data availability statement. This statement should provide the following information, where applicable:

- Accession codes, unique identifiers, or web links for publicly available datasets
- A list of figures that have associated raw data
- A description of any restrictions on data availability

Source data are provided with this paper. Mass spectrometry data are available in Supplementary Data 1. The mass spectrometry raw and MaxQuant output files were deposited to the ProteomXchange Consortium (www.proteomeXchange.org) via the PRIDE 66 partner repository with the dataset identifier PXD025055. Databases are available under the same dataset identifier. The original data generated in this study have been deposited in ZENODO repository server (https://zenodo.org/) with the dataset identifier 10.5281/zenodo.5752116. The accession code will be provided by the corresponding author upon reasonable request

Field-specific reporting

Please select the one below that is the best fit for your research. If you are not sure, read the appropriate sections before making your selection.

- [x] Life sciences
- [ ] Behavioural & social sciences
- [ ] Ecological, evolutionary & environmental sciences

For a reference copy of the document with all sections, see nature.com/documents/nr-reporting-summary-flat.pdf

Life sciences study design

All studies must disclose on these points even when the disclosure is negative.

Sample size	All experiments have been performed at least with 2 but more often with 3 and above biological replicates (see replication section below). However, some experiments have been performed two or three times under slightly different conditions (i.e. different mice lines, choice of fluorophore labelling or overexpression vs endogenous expression). The outcome of these experiments were all the time qualitatively the same. However, because of the variability of the experimental conditions we could not pool the data together. Therefore in some cases the number biological replicates included in this work is equal to one. Technical replicates (cells, granules) are generally above 20 insuring that the results are representative.
Data exclusions	For FACS, MS and western blot data, all measured samples were included in the analysis except for those experiments with technical failure during the process of the preparation. For imaging data (Fig. 1a, Fig. 1b, Fig. 1e, Fig. 2c, Fig. 2d, Fig. 3, Fig. 4, Fig. 6, Fig. 7, Supplementary Fig. 3, Supplementary Fig. 4, Supplementary Fig. 6 and Supplementary Fig. 7), cells that appeared unhealthy based on their morphology or granules that seemed not intact were excluded from analysis. Finally, bad quality images were not included in data analysis. The exclusion criteria were pre-established.
Replication	Fig. 1a, N=6; n=31 cells. Fig. 1b, N=1; n=33. Fig. 1c, N=1; n=2. Fig. 1d, N=3. Fig. 1e, N=3; n=20. Fig. 2b, N=1; n=4. Fig. 2c, N=1; n=3. Fig. 2d, N=1 IP; n=7 for IP6 and n=7 for IP8. Fig. 3a, N=2; n=21 for IP6 and n=19 for IP8. Fig. 3c, N=3; n=118 for IP6 and n=85 for IP8. Fig. 3e, N=3; n=74. Fig. 4, N=5 for IP6 and N=3 for IP8. Fig. 5a, N=1; n=3 for WT and n=6 for GzmB KI. Fig. 5b, N=1; n=14. Fig. 5c, N=1; n=16. Fig. 5d, N=1; n=16 for entire cells and n=35 for IS. Fig. 5e, N=3; n=275 for MCG and n=156 for SCG in 20 cells. Fig. 5f, N=1; n=18. Fig. 6b, N=1; n=52 for MCG and n=44 for SCG. Fig. 7a, N=3; n=16. Fig. 7c, N=3; n=25. Fig. 7d, N=3; n=17. Fig. 7e-f, N=1; n=300. Supplementary Fig. 1, N=6. Supplementary Fig. 2a, N=1. Supplementary Fig. 2b, N=1. Supplementary Fig. 2c, N=1. Supplementary Fig. 3, N=3; n=118 for MCGs and n=85 for SCGs. Supplementary Fig. 4, n=37 for MCGs and n=19 for SCGs. Supplementary Fig. 5, N=5 for IP6 and N=3 for IP8. Supplementary Fig. 6, N=1; n=200.<br>N= biological replicates (number of mouse preparation, cell culture or centrifugation gradient/IP), n= technical replicates or cells.<br>Detail information of N and n and statistics are provided in each figure legends and source data file.
Randomization	This study does not contain different groups that need randomization. Indeed no comparison between WT, KO or mutations was performed. Furthermore no comparison between treatment was done.
Blinding	Blind experiments were not performed as no comparison between groups were done.

Reporting for specific materials, systems and methods

We require information from authors about some types of materials, experimental systems and methods used in many studies. Here, indicate whether each material, system or method listed is relevant to your study. If you are not sure if a list item applies to your research, read the appropriate section before selecting a response.

Materials & experimental systems

n/a	Involved in the study
☐	☒ Antibodies
☐	☒ Eukaryotic cell lines
☒	☐ Palaeontology and archaeology
☐	☒ Animals and other organisms
☒	☐ Human research participants
☒	☐ Clinical data
☒	☐ Dual use research of concern

Methods

n/a	Involved in the study
☒	☐ ChIP-seq
☐	☒ Flow cytometry
☒	☐ MRI-based neuroimaging

Antibodies

Antibodies used	Detailed description is given in Table 1.
Validation	All the antibodies except anti-Sec61β antibody are commercially available and were well-established and tested for validation. The validation proof of in-house generated anti-Sec61β antibody is shown at the Source Data file (Supplementary Fig. 2a).

Eukaryotic cell lines

Policy information about cell lines

Cell line source(s)	P815 (ATCC® TIB-64™); NK92 (ATCC CRL 2407)
Authentication	The P-815 cell line was directly purchased from DSMZ (ACC1) and being authenticated (PO no. 8111044). NK92 cells were a kind gift from Prof. Persephone Borrow lab (University of Oxford) without being authenticated.
Mycoplasma contamination	The P815 cell line was not tested for mycoplasma contamination after receiving it from the company (ATCC). Same was true for the NK92 cell line.
Commonly misidentified lines (See ICLAC register)	This cell line is not misidentified

Animals and other organisms

Policy information about studies involving animals; ARRIVE guidelines recommended for reporting animal research

Laboratory animals	Synaptobrevin2-mRFP knock-in (Syb2 KI), granzyme B-mTFP knock-in (GzmB KI), double KI and WT mice of either sex were used at the age of 15-22 weeks. Animals were kept under the housing conditions of 22°C room temperature with 50-60% humidity and 12 h dark and 12 h light cycle.
Wild animals	None were involved in this study
Field-collected samples	This study did not involve samples collected from the field
Ethics oversight	Overall the study did not require specific ethic oversight. All experimental procedures were approved and performed according to the regulations by the state of Saarland (Landesamt für Verbraucherschutz, AZ.: 2.4.1.1). Ethical guidelines for the care and use of laboratory animals, issued by the German Government and approved by the Commissions for Institutional Animal Care and Use at Saarland University, Saarland, Germany, were followed (animal license number 41–2016) .

Note that full information on the approval of the study protocol must also be provided in the manuscript.

Flow Cytometry

Plots

Confirm that:

☒ The axis labels state the marker and fluorochrome used (e.g. CD4-FITC).

☒ The axis scales are clearly visible. Include numbers along axes only for bottom left plot of group (a 'group' is an analysis of identical markers).

☒ All plots are contour plots with outliers or pseudocolor plots.

☒ A numerical value for number of cells or percentage (with statistics) is provided.

Methodology

Sample preparation	T cells were isolated from spleen of laborartory mice.

Instrument	Cells were analyzed by BD FACSAriaIII analyzer .
Software	The data were analyzed by BD FACSDivaTM software and FlowJo v10.0.7 software.
Cell population abundance	The target effector cell population were from 85% viable T cells from culture and the high purity (97%) of effector cells were further analyzed for central memory and effector memory cells.
Gating strategy	The target effector cell population was gated from viable T lymphocytes based on their size and granularity. Effector Syb2 KI cells were gated based on mRFP signal from wiltype cells. The fluorescence signal of antibody stained cells were further analyzed by subtracting mRFP signal in unstained Syb2 KI cells. The immune cell subsets were then further analyzed according to the antibody labeling. Cells were gated according to positive and negative double staining of CD44 CD62L from viable cells of mRFP positive population from SybKI mice. This information can be fount in the material and method and Supp. Fig. 1.

☒ Tick this box to confirm that a figure exemplifying the gating strategy is provided in the Supplementary Information.
